# Influence of Intra-Articular Administration of Trichostatin A on Autologous Osteochondral Transplantation in a Rabbit Model

**DOI:** 10.1155/2015/470934

**Published:** 2015-03-18

**Authors:** Huacheng Hou, Ke Zheng, Guanghu Wang, Shiro Ikegawa, Minghao Zheng, Xiang Gao, Jinzhong Qin, Huajian Teng, Qing Jiang

**Affiliations:** ^1^Medical School of Nanjing University, Hankou Road, Nanjing, Jiangsu 210093, China; ^2^The Center of Diagnosis and Treatment for Joint Disease, Nanjing Drum Tower Hospital Affiliated to Medical School of Nanjing University, Zhongshan Road, Nanjing, Jiangsu 210008, China; ^3^Laboratory for Bone and Joint Disease, Center for Genomic Medicine, 4-6-1 Shirokane-dai, Minato-ku, Tokyo 108-8639, Japan; ^4^Orthopaedic Surgery, Centre for Orthopaedic Research, The University of Western Australia (M508), Crawley, WA 6009, Australia; ^5^Model Animal Research Center of Nanjing University, Xuefu Road, Nanjing, Jiangsu 210032, China

## Abstract

Autologous osteochondral transplantation (AOT) is a method for articular cartilage repair. However, several disadvantages of this method have been reported, such as transplanted cartilage degeneration and the lack of a connection between the grafted and adjacent cartilage tissues. To evaluate the effect of intra-articular administration of trichostatin A (TSA) on AOT, we conducted a case control study in a rabbit model. International Cartilage Repair Society (ICRS) macroscopic scores, the modified O'Driscoll histology scores, and real-time PCR were utilized to evaluate the results. At 4 weeks, both macroscopic and histological assessments showed that there was no significant difference between the TSA and control groups. However, the mean macroscopic and histological scores for the TSA-treated group were significantly higher than the scores for the control group at 12 weeks. TSA was shown to directly reduce collagen type II (COL2), aggrecan, matrix metalloproteinase (MMP), and a disintegrin and metalloproteinase domain with thrombospondin motifs 5 (ADAMTS-5) expression and to simultaneously repress the upregulation of MMP-3, MMP-9, and MMP-13 levels induced by interleukin 1*β* (IL-1*β*) in chondrocytes. In conclusion, TSA protects AOT grafts from degeneration, which may provide a benefit in the repair of articular cartilage injury.

## 1. Introduction

Articular cartilage injuries are one of the most common types of injuries observed in orthopedic diseases, and they can be caused by trauma, articular degenerative diseases, osteochondritis dissecans, hemophilia, and acromegaly. Lacking vasculature, injured articular cartilage has a limited ability to regenerate [1], and deep osteochondral lesions may contribute to the premature development of osteoarthritis [2].

Over the past 50 years, several clinical techniques have been developed to repair osteochondral defects, including drilling, microfracture, allogeneic osteochondral transplantation, AOT, autologous chondrocyte transplantation, and tissue engineering techniques. Specifically, AOT involves the transplantation of autologous osteochondral grafts from a nonweight bearing area to the lesioned area and can achieve satisfactory short-term clinical outcomes [3]. According to the current literature, up to 90% patients undergoing AOT experience alleviation of their clinical symptoms [4]. The disadvantages of this method include the limited donor area, donor site morbidity, the subsidence and degeneration of the graft, different mechanical properties between the transplanted and adjacent cartilage tissues, poor connection between the graft and adjacent cartilage tissues, and uncertain clinical long-term efficacy [5]. In addition, both magnetic resonance imaging (MRI) and histological evaluation have demonstrated postoperative cyst formation in the graft in some patients undergoing AOT [6–8], which may be an indirect indicator of cartilage degeneration. It is well known that inflammation plays a negative role in cartilage degeneration, and the suppression of inflammation may prevent the progression of graft degeneration.

Histone deacetylase (HDAC) inhibitors, a family of epigenetic regulators of gene expression, may potentially act as therapeutic agents for osteoarthritis. In animal models and in cell culture studies, HDAC inhibitors have exhibited promising anti-inflammatory abilities [9, 10]. It has been reported that TSA, an HDAC inhibitor, has anticancer [11] and anti-inflammation [12] effect, enhances autophagy [13], promotes stem cell differentiation [14], and improves embryonic development [15]. With respect to cartilage, TSA can block IL-1*α*- and oncostatin M-induced expression of MMPs and the ADAMTS protein family in human chondrocytes [16, 17]. Chabane et al. demonstrated that HDAC inhibitors could suppress the induction of nitric oxide and prostaglandin E2 synthesis, the expression of inducible nitric oxide synthase and cyclooxygenase 2, and proteoglycan degradation by IL-1 [18]. In adjuvant arthritis, TSA can effectively suppress synovial hyperplasia by inducing the expression of p16^INK4a^ and p21^Cip1^, two cell-cycle regulators in synovial fibroblasts [19]. Similarly, the subcutaneous administration of TSA can ameliorate synovial inflammation and suppress cartilage destruction in a collagen antibody-induced arthritis mouse model [20]. In an experimental osteoarthritis model in rabbits, TSA can prevent cartilage degeneration, including reducing hypocellularity and the loss of aggrecan [21].

We were interested in whether HDAC inhibitors could be a therapeutic option for AOT treatment. In the present study, we show that TSA inhibits graft degeneration following AOT but does not improve the integration between the grafted and adjacent cartilage tissues in a rabbit AOT model.

## 2. Materials and Methods

### 2.1. Animal Model

Animal experiments described in this research were approved by the institutional review board of Nanjing University. Human joint subjects were recruited with ethics review board approval and written informed consent from patients undergoing knee or hip replacement in the Center of Diagnosis and Treatment for Joint Disease, Nanjing Drum Tower Hospital Affiliated to Medical School of Nanjing University.

**Figure 1:**
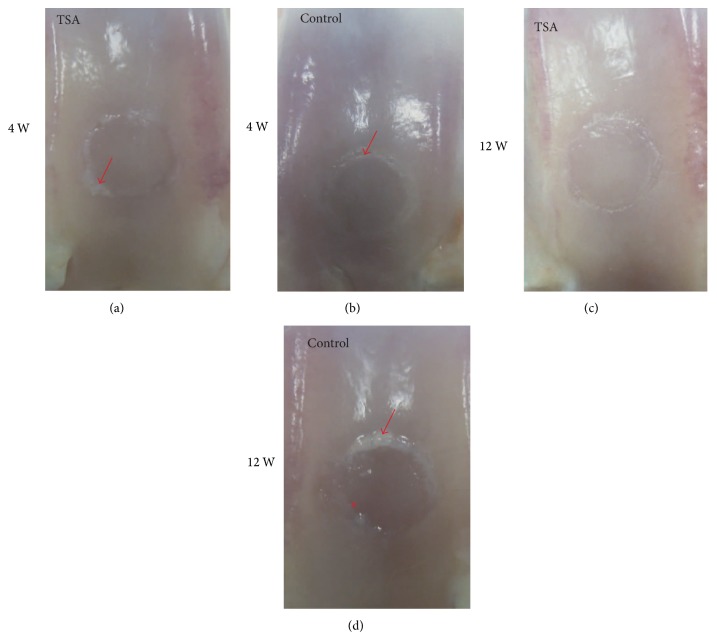
The macroscopic appearance of grafts in the trochlear groove at 4 weeks and 12 weeks after surgery in the TSA (a, c) and control groups (b, d) (red arrows indicate fibrillation around the grafts; a red asterisk indicates fissures above the grafts).

Twenty adult female New Zealand white rabbits (16 weeks old, average body weight of 2.5 kg) were obtained from the Animal Research Center of Drum Tower Hospital. They were kept in common rabbit cages and fed a standard diet with tap water ad libitum. After one week, each rabbit was anesthetized using ketamine and diazepam. After making a medial parapatellar incision in both knees, each patella was dislocated laterally, and an osteochondral defect (diameter, 3.5 mm; depth, 3 mm) was created on the patellar groove of the femur in both knees using a custom trepan. An osteochondral graft from one knee was filled into the defect site in the other knee (Figure 1). When the defect area was full, the patella was repositioned, and then the incision was closed by layered suturing. After surgery, the rabbits were returned to their cages and allowed to move about freely within their cages.

### 2.2. Treatment

On the day after operation, the rabbits were randomly assigned to two groups: the TSA treatment group and the control group. The TSA group was given intra-articular injections of 0.3 mL of TSA (Sigma-Aldrich, Saint Louis, MO, USA) in both knees once a week for 4 or 12 weeks. The concentration of TSA, as previously mentioned, was 250 ng/mL [21]. The control group was injected with the solvent of TSA, namely, 297 *μ*L of normal saline with 3 *μ*L dimethylsulfoxide (DMSO) (Sigma-Aldrich) under the same conditions. Rabbits were euthanized seven days after the last injection, and both knees were harvested. Simultaneously, macroscopic assessment was carried out with the ICRS macroscopic scores (Table 1).

### 2.3. Histological Grading

All of the samples from the control and TSA groups were fixed in 10% neutral-buffered formalin for 7 days, decalcified with 10% ethylenediaminetetraacetic acid (EDTA) (Sigma-Aldrich) at pH 7.4 for approximately 4 weeks, and then dehydrated in a graded ethanol solution series, embedded in paraffin, sliced consecutively into 5 *μ*m sections through the center of the graft, and stained with safranin O—fast green. The samples were graded based on the histological appearance of the graft and the adjacent tissue according to the modified O'Driscoll histology scores (Table 2) [5]. The other author independently reviewed the results of the histological scores for each sample, and the results were completely in agreement with the first scorer.

### 2.4. Chondrocyte Isolation and Culture

Patients from whom cartilage specimens were harvested all underwent total hip replacement for osteoarthritis. An expert chose an area of the replaced osteochondral block with an intact appearance as the specimen for cellular research. We obtained human chondrocytes according to the procedure published by Wang et al. [17]. Briefly, the cartilage specimens were digested overnight in Dulbecco's modified Eagle's medium: Nutrient Mixture F-12 (F12/DMEM) with 5% fetal bovine serum (FBS) and 0.08 mg/mL collagenase type II (Invitrogen, Grand Island, NY, USA). The liberated cells were washed with phosphate-buffered saline (PBS) and resuspended in F12/DMEM with 10% FBS and antibiotics (100 U/mL penicillin, 100 mg/mL streptomycin sulfate) (Invitrogen). Cells were seeded in tissue culture dishes at a concentration of 4 × 10^4^ cells/cm^2^ in F12/DMEM with 10% FBS. After 50% cell confluency was achieved, the concentration of FBS was reduced to 1%. Then, the cells were treated with TSA (250 ng/mL) with or without IL-1*β* (10 ng/mL) (R&D Systems, Inc., MN, USA) for 4 hours or 12 hours and harvested for downstream treatment.

### 2.5. RNA Isolation and Gene Expression Analysis

RNA was isolated from the upstream chondrocytes using TRIzol reagent (Invitrogen). First-strand cDNA was synthesized from total RNA using a PrimeScript RT reagent kit (Takara Bio, Otsu, Japan). Levels of mRNA expression were analyzed by real-time PCR with SYBR Green detection using an ABI PRISM 7500 Sequence Detection System (Applied Biosystems, Grand Island, NY, USA), and the expression values for each gene were calculated using individual standard curves normalized to the expression of glyceraldehyde 3-phosphate dehydrogenase (GAPDH). Gene-specific primer sequences are shown in Table 3, including the sequences for COL2, aggrecan, MMP-1, MMP-3, and MMP-13, and ADAMTS-5.

### 2.6. Statistical Analysis

Student's *t*-test was used to examine the differences between groups with respect to the ICRS macroscopic score, the modified O'Driscoll histology score, and gene expression levels. A value of *P* < 0.05 was regarded as a significant statistical difference. All of the data were independently processed by the coauthors and analyzed with the SPSS (Statistical Package for the Social Sciences) 17.0 system software (SPSS Inc., Chicago, Illinois, USA).

## 3. Results

All of the animals successfully underwent bilateral knee arthrotomy. No postoperative complications such as infection or disability were observed during the period of the test.

### 3.1. Macroscopic Findings

At the time of the second operation, the macroscopic assessment (Figures 1 and 4(a)) was carried out. At 4 weeks, the mean ICRS macroscopic score in the TSA-treated group and the control group was 9.8 ± 1.1 and 9.2 ± 1.1 (*P* = 0.47), respectively. The grafts in both groups showed a smooth cartilage surface with no fissures above them and less fibrillation around them. However, an obvious demarcation between the graft and the adjacent cartilage tissues was observed in all 12 cases. The synovium appeared to be normal in both groups. At 12 weeks, the mean ICRS macroscopic score in the TSA-treated group and the control group was 9.2 ± 0.7 and 8.2 ± 0.7 (*P* = 0.04), respectively. Compared with the control group, the grafts in the TSA-treated group showed smoother surfaces with less fibrillation around them and fewer fissures above them. However, the grafts in the TSA-treated group did not show any blurred demarcations with more connective tissue filling compared with the control group. The synovium appeared to be hyperplastic in the control group, possibly indicating that osteoarthritis was occurring.

### 3.2. Histological Findings

**Figure 2:**
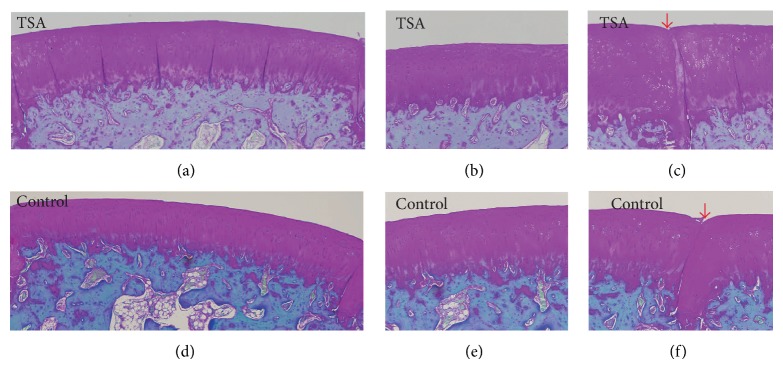
Histologic appearance of grafts in the trochlear groove at 4 weeks postoperatively in the TSA and control groups. (b) shows a magnified view of the part area in (a). (c) shows the gap between the grafted and adjacent cartilage tissues in (a). (e) shows a magnified view of the part area in (d). (f) shows the gap between the grafted and adjacent cartilage tissues in (d) (red arrows indicate the gap between the graft and the adjacent cartilage tissues).

**Figure 3:**
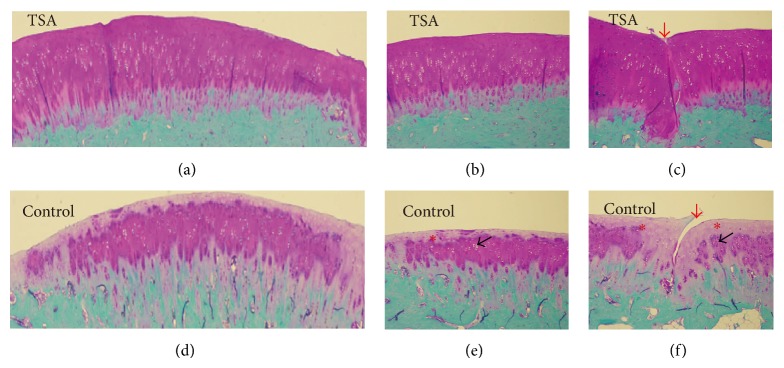
Histologic appearance of grafts in the trochlear groove at 12 weeks postoperatively in the TSA and control groups. (b) shows a magnified view of the indicated area in (a). (c) shows the gap between the grafted and adjacent cartilage tissues in (a). (e) shows a magnified view of the indicated area in (d). (f) shows the gap between the grafted and adjacent cartilage tissues in (d) (red arrows indicate the gap between the graft and the adjacent cartilage tissues, black arrows indicate hypocellularity, and red asterisks indicate loss of safranin O staining of the matrix).

**Figure 4:**
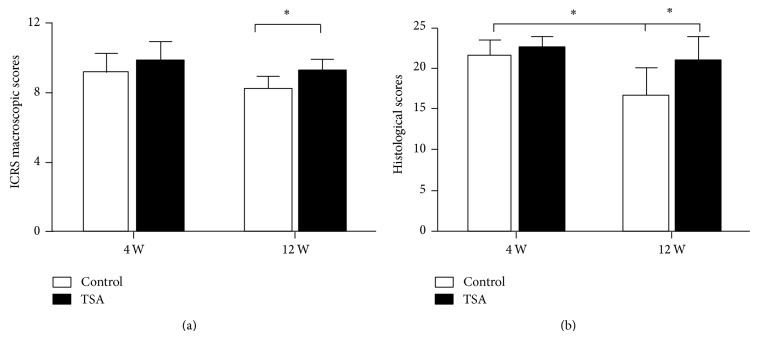
(a) ICRS macroscopic and (b) histological scores for the TSA and control groups at 4 and 12 weeks postoperatively. ^*^ *P* < 0.05 indicates a significant difference between the two groups.

At 4 weeks, histological assessment (Figures 2 and 4(b)) showed an improved mean total modified O'Driscoll histology score in the TSA-treated group compared with the control group (22.4 ± 1.2 vs. 21.5 ± 1.7), although the difference was not significant (*P* = 0.2). When stratified by each parameter, no significant difference was observed between the TSA-treated group and the control group. At 12 weeks, the mean total modified O'Driscoll histology score (Figures 3 and 4(b)) in TSA-treated group and the control group was 20.7 ± 2.9 and 16.7 ± 3.1 (*P* = 0.01), respectively. There were significant improvements in the following parameters: safranin O staining of the matrix, hypocellularity, and degenerative changes in the adjacent cartilage in the TSA-treated group compared with the control group. However, no significant difference was found with respect to bonding with the adjacent cartilage between the two groups at either 4 or 12 weeks.

### 3.3. TSA's Effects on Gene Expression

**Figure 5:**
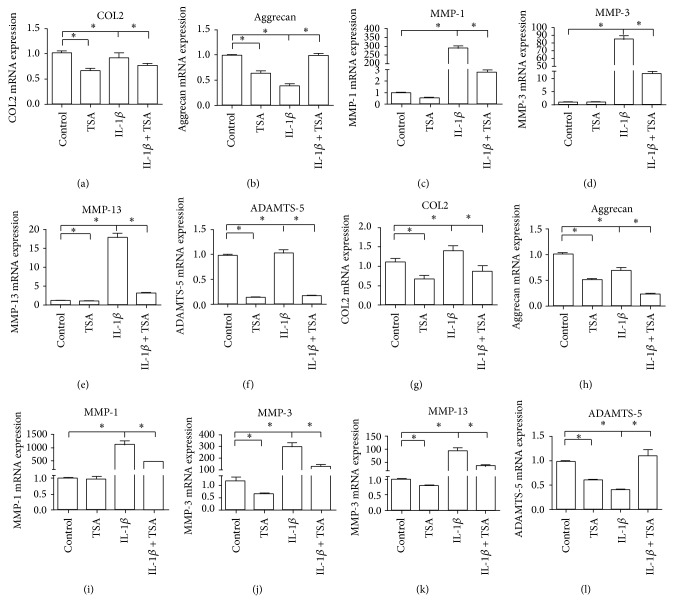
Effects of TSA on gene expression levels of anabolic and catabolic cartilage matrix factors in human chondrocytes. (a)–(f) represent cells that were treated with IL-1*β* and/or TSA for 6 h, and (g)–(l) represent cells that were treated with IL-1*β* and/or TSA for 24 h. ^*^ *P* < 0.05 indicates a significant difference between the two groups.

We explored the effect of TSA on the expression of anabolic (COL2 and aggrecan) and catabolic factors (MMPs and ADAMTS-5) in articular cartilage.  Figure 5 shows that TSA directly reduced the expression of COL2, aggrecan, MMPs, and ADAMTS-5 in culture at both 6 and 12 hours. After IL-1*β* treatment, the MMP-3, MMP-9, and MMP-13 mRNA levels were dramatically increased. In contrast, TSA treatment absolutely blocked IL-1*β*-induced MMP-3, MMP-9, and MMP-13 expression.

## 4. Discussion

While using AOT to treat osteochondral defects is very common in clinical practice [22–24], accumulating evidence from basic research to clinical observation has given orthopedists reason to pause. Implanted cartilage degeneration and loss as well as subchondral cyst formation have often been found on medium- to long-term follow-up in patients [25, 26]. Degeneration of the transplanted cartilage and the lack of a connection between the graft and the adjacent cartilage tissues have been observed in both animal models and human specimens [8, 27–30]. In in vivo experimental study, Smyth et al. found improvements in both the macroscopic and the histological appearances of grafts treated with platelet-rich plasma (PRP), and the anti-inflammatory effects of PRP were considered to play a key role in this procedure [29], indicating that suppressing inflammation may be a new approach to treat graft degeneration after AOT.

It is well documented that inflammatory cytokines, which upregulate the expression of catabolic genes in chondrocytes, play a crucial role in the pathological process of articular cartilage degeneration [31]. Similarly, there is evidence that surgical trauma can increase IL-1*β* and tumor necrosis factor-*α* (TNF-*α*) levels, leading to upregulation of MMP and ADAMTS family expression [32, 33]. TSA has been proven to reduce ADAMTS-5 levels and inhibit the IL-1-mediated upregulation of MMP expression in cultured chondrocytes. Furthermore, Chung et al. reported that TSA can also suppress the expression of TNF-*α*, which mediates the upregulation of MMP and ADAMTS family expression in adjuvant arthritis [19]. In the present study, our results showed that greater safranin O staining, less hypocellularity of the grafted cartilage, and less degenerative change in the adjacent cartilage occurred in the TSA-treated group compared with the control group. While TSA did significantly suppress the IL-1*β*-induced expression of several MMPs and ADAMTS-5, the finding that TSA also downregulated the expression of COL2 and aggrecan in cultured chondrocytes was unexpected. Therefore, our study showed that TSA may protect transplanted cartilage from degeneration primarily through its anti-inflammatory properties. At present, more and more researchers hope to eliminate treatment obstacles through tissue engineering. However, tissue engineering cannot prevent graft degeneration. The combination of tissue engineering with TSA treatment may provide a promising approach for researchers.

## 5. Conclusions

This study shows that TSA may have tremendous potential in the treatment of graft degeneration after AOT, although it does not improve the integration between grafted and adjacent cartilage tissues in a rabbit model. Our results may lead to a new therapeutic strategy to improve clinical outcomes after AOT.

## Figures and Tables

**Table 1:** The ICRS macroscopic score.

Characteristic	Grading	Score
Degree of defect repair	Level with surrounding cartilage	4
	75% repair of defect depth	3
	50% repair of defect depth	2
	25% repair of defect depth	1
	0% repair of defect depth	0

Integration to border zone	Complete integration with border zone	4
	Demarcating border <1 mm	3
	3/4 of repair tissue integrated, 1/4 with notable border >1 mm	2
	1/2 of repair integrated with surrounding cartilage, 1/2 with a notable border >1 mm	1
	From no contact to 1/4 of repair integrated with surrounding cartilage	0

Macroscopic appearance	Intact smooth surface	4
	Fibrillated surface	3
	Small, scattered fissures or cracks	2
	Several, small, or few but large fissures	1
	Total degeneration of defect area	0

**Table 2:** The modified O'Driscoll histology score.

Category	Score
Nature of predominant tissue	
Cell morphology	
Hyaline cartilage	4
Incompletely differentiated mesenchyme	2
Fibrous tissue or bone	0
Safranin O staining of the matrix	
Normal or nearly normal	3
Moderate	2
Slight	1
None	0
Structural characteristics	
Surface regularity	
Smooth and intact	3
Superficial horizontal lamination	2
Fissure 25–100% of the thickness	1
Severe disruption	0
Structural integrity	
Normal	2
Slight disruption, including cysts	1
Severe disintegration	0
Thickness	
100% of normal adjacent cartilage	2
50–100% of normal cartilage	1
0–50% of normal cartilage	0
Bonding to adjacent cartilage	
Bonded at both ends of graft	2
Bonded at one end or partially at both ends	1
Not bonded	0
Freedom from cellular changes of degeneration	
Hypocellularity	
Normal	3
Slight	2
Moderate	1
Severe	0
Chondrocyte clustering	
No clusters	2
<25% of the cells	1
25–100% of the cells	0
Freedom from degeneration changes in adjacent cartilage	
Normal cellularity, no clusters, normal staining	3
Normal cellularity, mild clusters, moderate staining	2
Mild or moderate hypocellularity, slight staining	1
Severe hypocellularity, poor or no staining	0

**Table 3:** Gene specific primer sequences for real-time PCR.

Gene	Primer sequences
COL2	Forward: 5′-TGGACGATCAGGCGAAACC-3′
	Reverse: 5′-GCTGCGGATGCTCTCAATCT-3′

Aggrecan	Forward: 5′-ACTCTGGGTTTTCGTGACTCT-3′
	Reverse: 5′-ACACTCAGCGAGTTGTCATGG-3′

MMP-1	Forward: 5′-AAAATTACACGCCAGATTTGCC-3′
	Reverse: 5′-GGTGTGACATTACTCCAGAGTTG-3′

MMP-3	Forward: 5′-AGTCTTCCAATCCTACTGTTGCT-3′
	Reverse: 5′-TCCCCGTCACCTCCAATCC-3′

MMP-13	Forward: 5′-ACTGAGAGGCTCCGAGAAATG-3′
	Reverse: 5′-GAACCCCGCATCTTGGCTT-3′

ADAMTS-5	Forward: 5′-GAACATCGACCAACTCTACTCCG-3′
	Reverse: 5′-CAATGCCCACCGAACCATCT-3′
